# Surgical Memoranda by M. Vidal

**Published:** 1845-03

**Authors:** 


*Surgical Memoranda, by M. Vidal.*—1. *Luxation of the First Phalanx of the Thumb.*—The reduction of this accident is almost always extremely difficult. A variety of suggestions have accordingly been proposed to effect the object in view. Shortly after I arrived (says our author) in Paris, a case of dislocation of the thumb backwards was brought into the Hotel Dieu: every imaginable attempt to reduce the displacement was ineffectually tried. M. Dupuytren delivered a lecture upon the accident, and endeavoured to prove that the irreducibility was caused by the altered position of the lateral ligaments of the joint; these bands, in consequence (he supposed) of having become stretched in an oblique direction, bound down the digital bone against its metacarpal fellow. Upon that occasion, I showed that the real obstacle was a *boutonniere* of muscular substance, which strangulated the head of the metacarpal bone, and became the more and more tightened in proportion to the force with which traction was applied to the displaced phalanx. This impediment is formed on the outer side of the external portion, and on the inner side by the internal portion, of the small *flexor pollices* and *adductor brevis.* As the heads of these muscles are inserted into the upper extremity of the first phalanx, they are necessarily forced backwards along with the dislocated bone, and the upper metacarpal protuberance is thus held fast between them. To effect the reduction tractions in a variety of directions had been (as already mentioned) tried for a length of time; but all in vain. I proposed to cut away the head of the metacarpal bone; but the suggestion was not, and perhaps wisely, adopted. M. Malgaigne has more judiciously suggested that it would be better to divide the external portion of the *boutonniere* or strangulating muscular band.—*Ibid.*

